# Medical and Physical Intelligence

**Published:** 1824-03

**Authors:** 


### MEDICAL AND PHYSICAL INTELLIGENCE.

### PHYSIOLOGY.

1. *Further Experiments on various Parts of the Encephalon; by* M. FLOURENS.—THIS industrious physiologist has recently communicated to the Institute several successive Memoirs, giving a detail of new experiments on the brain and nerves. Into these we cannot enter fully, but lay before our readers the general results.

It appears then, 1st. that, "une dose determinée," opium acts exclusively on the cerebral lobes; belladonna on the tubercula quadrigemina; and alcohol on the cerebellum.

2d. That the physical results of the action of each of these substances on the relative parts, are absolutely the same as those produced by mechanical lesion of the parts in question. Thus, for instance, when the substance only acts upon the cerebral lobes, the functions of these lobes alone are impaired: when they act on the cerebellum, its functions only suffer; and, when on the tubercula quadrigemina, their functions, &c.

3d. That the action of each substance always leaves after death, and even determines during life, certain traces which serve to point out the organ which is affected.

4th. That camphor, the ethers, &c. act in a manner analogous to alcohol; the extracts of hyoscyamus, of the lactuca virosa, &c. in a manner similar to opium.

These memoirs have been sent to a commission, composed of MM. CUVIER, DE HUMBOLDT, PORTAL, DULONG, and DUMERIL, whose report we shall not fail to lay before our readers on a future occasion.—(*Revue Medicale.*)

2. *Respiration of the Fœtus.*—M. GEOFFROY-SAINT-HILAIRE has lately communicated to the Academie des Sciences a Memoir on this subject. This distinguished naturalist, proceeding upon the principle that there is no organisation without the combination of a fluid capable of being assimilated, and no assimilation without oxygenation, or previous respiration, has endeavoured to demonstrate—

1st. That there exists a respirable gas in the waters of the amnios, the presence of which seems to be indicated by the experiments of MM. Chevreuil and Lassaigne.

2d. That the fœtus does, in the same manner as aquatic insects, separate the air contained in the circumambient fluid by all its pores, like so many tracheæ; and, further, that it brings into contact with the air thus obtained the venous blood, which circulates in the minutest capillaries of the dermis.

3d. That the contraction of the womb and abdominal muscles produce a sufficient degree of compression to effect this phenomenon during ordinary respiration.

3. *Peculiarity with respect to the Teeth of the Guinea Pig.*—M. Rousseau, in a letter addressed to the Royal Academy of Medicine, asserts that the guinea-pig loses the first *dens molaris* four or five days before its birth, and that it is absorbed in the uterus. The animal is born with the teeth already developed, and sufficiently strong for the purpose of chewing.—(*Bulletin des Sciences Medicales.*)

4. *Experiment with Laudanum on a Dog.*—In corroboration of the experiments of Mr. Jukes, I beg to state that I injected an ounce of laudanum into the stomach of a dog, aged seven weeks; and, in twenty minutes afterwards, when the dog staggered and became dull and heavy, I injected a quart of warm water into the stomach, and withdrew therefrom full as much of the liquid contents of that viscus, in which the smell and taste of laudanum was strongly perceptible. The dog was lively after the operation, and did perfectly well, without employing any other means. The instrument used was sent me by Mr. Gill, and is an improvement on the one suggested to him by Mr. Jukes: it consists of a pewter syringe, (which will contain nine ounces of fluid,) with leather sucker, kept oiled, brass pipe and stop-cock, which is readily applied to a gum-elastic tube; and at the lower end of this tube is a small ball of ivory, perforated, through which the warm water is injected, and the liquid contents of the stomach withdrawn. The operation was easily and quickly performed, the dog remaining perfectly quiet at the time. Position made no difference in respect to the operation: the fluid contents of the stomach were as easily withdrawn, when the dog was held up by one of my pupils, (Mr. Miles,) the hinder feet only touching the floor, as when laid on the left side.—(*Letter from* Mr. Yeatman, *of Frome.*)

---

### MORBID ANATOMY AND PATHOLOGY.

5. *Cancer of the Stomach, with Perforation of the Vena Portæ.*—A subaltern officer, fifty-one years of age, of a lymphatic temperament, entered the military hospital of Val-de-Grace for a diarrhœa, which lasted six months. He was thin, pale, and much emaciated; his extremities were cold, and a tumor was perceptible in the epigastric region. At the end of twenty days, during which time he appeared to be recovering, from the use of farinaceous diet, constipation supervened, with tympanetic enlargement of the abdomen, frequency of pulse, heat of skin, shivering, and death. On opening the body, much water was found in the peritoneal sac, while the stomach, gastro-hepatic epiploon, pancreas, commencement of the duodenum, the subjacent cellular tissue, and the vena portæ, were united into one mass; the cavity of the stomach was filled with black blood, and presented a carcinomatous appearance towards the pylorus, occupying four inches in breadth, an ulceration in the centre of which opened a communication into the vena portæ, from whence the blood in the stomach had no doubt been derived.—*(Bullet. de la Societé Philom.)*

6. *Case of Fistulous Opening into the Trachea.*—Examples of wounds involving the trachea are not uncommon, and, when the head is maintained in a proper position, viz. bent upon the breast, such injuries frequently do well. But little information, however, is to be found in practical writers respecting the nature of such wounds, when the healing is left to the unassisted efforts of nature. A case of this kind is thus described by M. Bienvenu, a French naval surgeon of the first class:—"A company's ship, which we captured near Ceylon, was armed with English, Indians, and Chinese. Among the last was one about twenty-five or thirty years of age, in whom we remarked a peculiarity worthy of notice. At the anterior part of the neck, and beneath the thyroid cartilage, was situated a transverse opening, not unlike the mouth; the edges of it were vermilion, and perfectly cicatrised; they might easily have been mistaken for lips. He informed us that this opening was the effect of a cut with a knife, which he had received many years before, and that he experienced no other inconvenience than that of being obliged to bring the lips of the aperture together, so as to close them accurately, whenever he wanted to swallow any thing; and that he could neither sing nor speak if it was not perfectly shut: but, as to the rest, that he breathed more freely by this opening than by the mouth or nose. By means of this aperture, we could easily see the interior of the trachea and bronchia, and even the inferior opening of the glottis. He introduced different bodies into the trachea in our presence, and moved them about in every direction, without experiencing any acute pain.

"It is easy to conceive that this man could neither sing nor speak when this opening into the windpipe gaped; but it is not quite so easy to understand the difficulty which he experienced in deglutition: yet any one may satisfy himself that this act is impeded and difficult, or even impracticable, when the chin is raised, while it is accompanied with ease when the head is approximated to the chest. In the former case the larynx is elevated with difficulty, while the reverse happens in the latter."

This case goes to prove that the mucous lining of the trachea is not gifted with the same sensibility as that of the larynx; a fact, we believe, pretty well ascertained, though not often illustrated by such direct evidence.—*(Journal Universel des Sciences Medicales.)*

7. *On the Red Disease (Mal Rouge) of Cayenne.*—Dr. BERGERON, of the French navy, has lately published a description of this disease, which bears some analogy to elephantiasis. It attacks equally both sexes, most commonly blacks and mulatoes, those especially who live upon fish or unwholesome provisions, and very seldom affects the white inhabitants; it is endemic in that climate, and has its seat in the skin and cellular texture beneath it. This disease first shows itself by red patches, not circumscribed, having a great disposition to extend themselves; afterwards turning yellowish, and lessening the sensibility of the part in which they occur; in which respect it bears some resemblance to elephantiasis, lepra, and the Barbadoes disease.

The spots show themselves most commonly on the shoulders, forehead, nose, and ears, afterwards spreading to the back, thighs, and feet; that is, the parts most exposed to the action of the sun and the air suffer first. At this stage of the disease the health does not suffer, and it often remains for a long time stationary: at length, however, the lips, cheeks, forehead, and eyelids, swell, and become hard; the heat of the skin is augmented; the voice is hoarse, but the internal organs do not appear to suffer. The second stage of the malady commences with an eruption of hard and insensible tubercles, or sometimes small pustules; the skin becomes wrinkled and uneven; the breath is fetid, and the patient complains of lassitude. The third stage of the disease is marked by violent fever and thirst, loss of appetite, deep ulcerations and disorganisation of the skin; the loss of the hair, nails, or even of the nose and ears; and affections of the digestive organs.

M. Bergeron endeavours to prove, notwithstanding some marks of similarity, that this disease is essentially different from the elephantiasis; and observes, that those organs that most commonly suffer in the one case remain unaffected in the other: nevertheless, they seem to differ more in degree than in kind. Among the causes which the author assigns for the great frequency of this disease among the blacks, is the absorption of the solar rays, and their constant exposure to suppressed perspiration from the breezes of the country. When this complaint attacks in an acute form, its rapidity is frightful.

Our author does not say much as to the mode of treatment: he justly condemns the separation of these unfortunate beings from society, under the dread of contagion; they are generally, it seems, abandoned to themselves. M. Bergeron condemns the oxymuriate of mercury as an external application, but thinks its internal exhibition might answer better. He considers cauterisation as the most appropriate remedy at the commencement, especially the employment of the nitrate of silver. —(*Journal Universel des Sciences Medicales,* Decembre 1823.)

---

### THERAPEUTICS AND MATERIA MEDICA.

8. *Hydrophobia, and Injection of warm Water into the Veins.*—Our readers may recollect hearing of this experiment, performed by M. MAGENDIE on a baker, who was brought into the Hotel Dieu, labouring, as it was supposed, under symptoms of hydrophobia, and who had moreover a wound on the hand. M. Magendie, in spite of the terrible convulsions with which the man was affected, threw about a pint of warm water into the veins of the patient's arm, and in half an hour the relief obtained was very considerable; inasmuch as he could drink, the convulsions had ceased, and he had no longer any disposition to bite: however, he died on the sixth day; and we only mention the case here because it appears that the man had never *been bitten at all*, and that the wound of the hand was produced by his having fallen upon a broken cup.—(*Revue Medicale*, Novembre.)

9. *Tincture of Nicotiana in Ischuria.*—Dr. WESTBURG, of Halmstad, in Sweden, has derived great advantage from the use of tincture of nicotiana in the treatment of ischuria: he gives twenty drops every hour, in a glass of linseed tea, and he has universally observed the most beneficial results, sometimes even from the second or third dose. He also employs this remedy successfully in blenorrhœa, when the patient can only make water drop by drop.—(*Idem.*)

10. *On the Use of the Actæa Racemosa in Phthisis Pulmonalis.*—The following account of the effects of this drug is given by Dr. T. S. GARDEN, of Charlotte. We regret that he does not mention either the dose of the medicine or the mode of its exhibition.

"I am probably the only physician who has ever used it in his own person, or who has any knowledge of its virtues and effects in disease, except those in my immediate section of country. This medicine has been in common use with the vulgar, in many parts of the western country, for some time past, as a remedy, and I have for a considerable time laboured under a conviction of its efficacy, from actual experiment upon my own person: I have, however, delayed giving it publicity until other evidence could be adduced, lest I should be thought the boasted discoverer of a remedy, which had no claim to merit except in the imagination of its inventor. I design merely to state facts, which can be supported by the testimony of those physicians who were acquainted with my own situation, and of those gentlemen who, in conjunction with me, have witnessed its effects upon others. I can ascribe the degree of health which I enjoy at present to nothing but the use of this medicine, aided by suitable regimen; and nothing but utter despair and entire extinction of all hope of recovery, together with a want of confidence in all others, induced me, upon the bare testimony of vulgar report, to hazard the experiment. In a short time my estimation of its virtues were greatly increased, and the expectations which had been excited of its ultimate success were finally realised. Shortly after commencing the use of this medicine, the hectic paroxysms, which had attended me for some time previous, were entirely checked; the nocturnal evacuations from the surface of the body, to which persons affected with phthisis are subject in the secondary stages, began to diminish; the expectoration of a fluid from the vessels of the lungs and bronchia, resembling pus in appearance, was speedily arrested; the cough became much less troublesome and less frequent; my pulse, which for some time before had never been lower than from 100 to 120 pulsations in the minute, was reduced to the medium standard; the pain in my right breast and side left me; my strength and appetite began to improve; and I speedily abandoned the use of all medicines, or any other means, except attention to regimen and exercise. A period of twelve months or more had elapsed, from my primitive ill health to using this medicine, during which time I had bled freely and copiously, kept up a constant discharge from the surface of my breast, by the use of blisters, setons, &c. and adhered strictly to a vegetable regimen, but without any relief.

"It was the opinion of the celebrated Dr. Rush, that, if there existed a remedy for hectic fever, it was a tonic, and that it belonged to the vegetable kingdom; and such is evidently the nature of the *actæa racemosa.* Like the digitalis, it disorders the sensorium, and operates in a powerful manner upon the secreting and absorbent systems. When exhibited in a full dose, it prostrates in a distressing degree, producing nausea, vertigo, pains of the extremities, anxiety, dilatation of the pupil, quick small pulse, with universal restlessness and uneasiness. These effects are immediate and transitory; its ultimate and remote operation is the converse of the above. It is this which gives it the supremacy over all other medicines of the same class."—*(American Med. Recorder.)*

### SURGERY.

11. *Case of Imperforate Anus in a Female.*—In January last, (says Dr. Sharpless,) I was called, during my tour of duty in the Dispensary, to see a female child, three weeks old, with imperforate anus, which had not been discovered till the infant was ten days old. On examination, I found the situation of the anus closed by a thick dense membrane, the fæces passing forward into the vagina, through an opening the size of a quill. By the probe I could discover that the rectum was quite enlarged to about half an inch of where the anus should be, and the stools having a free passage forward. The child, from birth, had been subject to great tenesmus, which perhaps arose from the tortuous course of the excrements.

In the presence of my friends, Drs. J. K. Mitchell and S. M. Fox, I introduced a small trocar, much curved, through the opening into the vagina, and protruded the stilet through the closing membrane, which was exceedingly tough and hard, requiring great force to accomplish it. The opening thus formed was enlarged to the size of a goose-quill by the bistoury, and a tent placed in, with directions to the parent to withdraw it as soon as an inclination to stool took place, which could always be foretold by the straining. This was done; but the opening was so small, and the fæces being determined forward by a ledge of flesh, little passed.

The object intended was to dilate the opening by tents; but this plan was soon found unavailing, and with a bistoury I divided this ledge and enlarged the whole passage, forming an unobstructed outlet of the natural size.

I now introduced a piece of the largest stomach tube, two inches in length, and extending far above the opening into the vagina. This tube was wrapped with bougie plaster to a considerable size, opposite the forward passage, to prevent any matters going that way. This was withdrawn every day, when no disposition to stool existed, and cleaned, and immediately returned. The bowels were kept laxative by castor-oil, and the fæces all passed through the tube. The irritation of the foreign body soon subsided, the tenesmus disappeared, and in two months the opening through the recto-vaginal septum was closed. The tube was now left out several hours every day, so that any sphincter that might exist should be called into action. In a short time a natural contraction seemed to take place; the edges of the wound became callous and cicatrised;, and in four months the child was perfectly well, presenting such an appearance that no person, ignorant of the case, could, upon the most minute examination, discover that any malformation had ever existed.—*(Philadelphia Journal.)*

12. *Case of Imperforate Anus in a Male, in which an Operation was performed by* M. Wolff.—The subject of this operation was a male infant, who came into the world of a weak form and unhealthy hue, altogether resembling a seventh or eighth months' child. The imperfection in the anus was not discovered till the evening of the twelfth day from his birth: during this time he was restless, cried much, and was at last affected with hiccup and convulsions. M. Wolff, on being called in, found the abdomen protuberant, hard, and painful to the touch; there were also nausea and vomiting, with great depression of strength. He instituted an examination, and discovered the cause of the symptoms. Next day he performed the operation, by pushing in a large lancet into the middle of the perineum, a few lines distant from the os coccygis; he passed it an inch deep towards the sacrum without meeting with the rectum. After stopping the bleeding he renewed the puncture, but without effect, although nearly two inches deep. He felt, however, the bladder through the wound, and also the hypogastric arteries pulsating. He had now recourse to a pharyngotome, with a bent and somewhat broad tube. This was pushed fully two inches deep, and entered the rectum; a clyster was immediately administered, which brought away some meconium. By the use of clysters and setons retained in the wound, the child rapidly recovered, so that, by the seventh day from the operation, the discharge per anum followed naturally; and, when seen some time afterwards, it was in good heath, and free from any disturbance in the functions of the rectum. In cases where the imperforation is of the kind described, M. Wolff prefers the pharyngotome to the trocar and lancet.—(Langenbeck's *Neue Bibliothek für die Chirurg. Philadelphia Journal.)*

We lately saw a child, on whom our friend, Mr. C. Hutchison, operated for imperforate anus with success; that is, so far as concerned the operation. This gentleman has operated in several similar cases; but we forbear entering into particulars, as there is a probability of the public becoming acquainted with them through a different channel.

---

### MIDWIFERY.

13. *Inversion of the Uterus.*—Dr. Teallier relates the following case. Madame R—, twenty-four years of age, well formed, became pregnant five years after her marriage; she experienced great chagrin during the course of her pregnancy, but completed her full period of gestation without any accident. On the 2d of September, 1823, Mad. R. was delivered safely, after a labour which lasted thirty-six hours; the placenta was extracted shortly afterwards without difficulty, and the patient was put to bed cautiously, and expressing herself comfortable. The next day the patient felt pain and tension in the abdomen; the bowels were costive, and the urine was scanty, and produced pain in voiding it. By the 7th of the month all these symptoms were removed, and every thing went on satisfactorily until the 12th, when Dr. T. was called at one o'clock in the morning to Madame R., who, in making violent efforts to evacuate the contents of the rectum, had felt a bulky mass of flesh descend through the vagina, which, though it did not produce much pain at the time, in about an hour after was followed by great pain in the belly and the displaced parts, and in the groins, with strong efforts to vomit and a sensation of faintness. She was much alarmed, and retired to bed, where she was lying, supporting between her thighs a smooth tumor, of a deep red colour, of the size and shape of a large pear; its large extremity was resting on the thighs, its pedicle was tied within the labia. There was no doubt of the nature of the case, and Dr. T. proceeded to the reduction, first, by placing the patient in a proper posture, and then returning the tumor, with his right hand well oiled, into the vagina; but, in endeavouring to restore it to its natural situation, the hardness and contraction of the neck rendered it impossible, the sensibility of the organ being so much augmented that the least pressure produced violent pain. Dr. T. therefore suspended his attempts, determined to renew them when circumstances were more favourable. The patient was placed upon her back, the pelvis elevated, the thighs closed, and emollient fomentations and injections were employed; a rigid diet was observed for the first twenty-four hours. There was no hemorrhage, but little pain in the abdomen or fever.

On the evening of the 13th, an obstinate cough, attended with fever and some pain in the abdomen, came on, but a large bleeding on the 14th removed these symptoms; and on the 18th, the condition of the patient continuing to be satisfactory, Dr. T., finding the tumor softer and smaller, determined to make another attempt at reducing it to its natural situation, and which he succeeded in effecting in one hour and a half, making during that time a moderate and continued pressure with the hand. No symptoms of consequence ensued, that could be ascribed to the accident; and, notwithstanding a slight attack of phlegmasia dolens, she recovered perfectly five weeks after her accouchement.—(*Journal Universel*, Novembre.)

### CHEMISTRY.

14. *Eritrogene.*—A new principle, to which the name of Eritrogene has been given, is described by Sig. Bezio. In examining the body of a person who died of jaundice, in the hospital at Venice, a fluid was found in place of the bile, which bore none of the characters of that secretion, and which was given to Sig. Bezio, who read an account of it to the Atheneum at Venice. The contents of the gall-bladder were not homogeneous, but consisted of a clot of filaments in an adhesive fluid, about as thick as honey: the liquid portion was purple, and the clot white, with black and red spots; the smell resembled that of putrid fish. By washing, the soluble portion was separated; it was then heated with water, and agitated, by which means the adipose portion separated, and collected on the surface. It was removed when cold, and dried on blotting-paper. It was of a greenish colour, and had the odour of bile. The fibrous matter collected at the bottom of the vessel was found to be fibrine, but little altered; the fatty matter, on examination, yielded stearine and elaine: the part which remained undissolved was of a fine green colour, and, when boiled in fresh alcohol, formed a green solution, which, by partial evaporation, yielded rhomboidal crystals of an emerald green. This is Eritrogene. The substance so named is transparent, flexible, unctuous, has the odour of putrid fish, but no taste. It fuses at 110° of Fahrenheit, and, if heated to 122°, it becomes volatilized, yielding, in contact with the atmosphere, a purple vapour: it is not soluble in ether or water, but is readily dissolved in alcohol. It has a great affinity for nitrogen; and the azotated eritrogene is said to be precisely the same as the colouring matter of the blood. Bezio further remarks, that eritrogene, or something very like it, exists in the chyle; and conjectures that, when it reaches the lungs, nitrogen is absorbed as well as oxygen, and colour thus given to it.—(*Journale de Fisica*, No. vi.)

---

MISCELLANEOUS.

15. *Inoculation with Syphilitic Matter.*—Three students of medicine in Paris, having adopted the theoretical opinion that the existence of any specific virus was but an antiquated and absurd doctrine, lately resolved to illustrate the new discovery on their own persons. They inoculated themselves with matter from venereal sores, by means of punctures in the arm with a lancet, resembling those usually practised in vaccinating. In one instance, the operation was followed by enlargement of the glands of the axilla, and was treated by simple antiphlogistic means, agreeably to the opinion already mentioned of its nature. Nevertheless, the swelling continued to increase,—ran on to suppuration, destroyed a considerable portion of the axilla,—and is not yet healed.

In a second, the puncture became inflamed and ulcerated; a chancre formed, which had all the characters of a true venereal sore, [query, what may these be?] and was treated, as in the preceding case, by antiphlogistics. The ulceration continued to spread in an alarming manner, until at length the young man was induced to consult an eminent practitioner, who informed him that the sore was syphilitic, and absolutely required the use of mercury. The unfortunate subject of this experiment put a period to his existence by opening the crural artery.

We copy this case, not because we agree with the writer in regarding it as calculated to throw any additional light on the nature of syphilis, but that it may serve as a caution to other students who may be ambitious of distinguishing themselves by the performance of similar experiments on their own persons, without having strength of mind to bear their loathsome and disgusting results.—(*Gazette de Santé*, Dec.)

16. *Hunterian Society.*—The fifth annual meeting of the Hunterian Society was held at the Society's room, 18, Aldermanbury, on Wednesday, February 4th; when the following members were elected officers for the year ensuing.

*President*—William Babington, M.D. F.R.S.

*Vice-Presidents*—H. Lidderdale, M.D. Benj. Travers, Esq. F.R.S. John Vetch, M.D. and William Compson, Esq.

*Treasurer*—B. Robinson, M.D.

*Secretaries*—J. T. Conquest, M.D. F.L.S. and Wm. Cooke, Esq.

*Council*—B. G. Babington, Esq. Thos. Bell, Esq. Thos. Callaway, Esq. W. D. Cordell, Esq. R. Dunglison, Esq. James Hamilton, M.D. John Hooper, Esq. J. C. Knight, Esq. Lewis Leese, Esq. J. Miles, Esq. J. Roberts, Esq. and J. H. Spry, Esq.

From the Report, it appeared that the number of the Society's members had increased within the year; and the balance in the treasurer's hands was about one hundred pounds.

---

### MONTHLY CATALOGUE OF MEDICAL BOOKS.

### METEOROLOGICAL JOURNAL,

*From January 20, to February 19, 1824.*

By Messrs. William Harris and Co. 50, Holborn, London.

| | Moon. | Rain gauge | Therm. | | | Barom. | | De Luc's Hyg. | | Wind. | | Atmospheric Variation. | | |
|---|---|---|---|---|---|---|---|---|---|---|---|---|---|---|
| | | | 9 A.M. | Max. | Min. | 9 A.M. | 10 P.M. | 9 A.M. | 10 P.M. | 9 A.M. | 10 P.M. | 9 A.M. | 2 P.M. | 10 P.M. |
| Jan. | | | | | | | | | | | | | | |
| 20 | | | 40 | 42 | 38 | 30.00 | 29.88 | 90 | 90 | S | WSW | Foggy | Foggy | Overc. |
| 21 | | | 40 | 42 | 39 | 29.55 | 29.40 | 90 | 92 | SW | WSW | Fine | Rain | Rain |
| 22 | | | 42 | 43 | 40 | 29.20 | 28.85 | 87 | 92 | WSW | SW | Rain | | |
| 23 | ☾ | | 43 | 43 | 40 | 28.70 | 29.94 | 85 | 93 | W | SW | Fine | Fair | |
| 24 | | | 42 | 45 | 40 | 29.67 | 29.74 | 83 | 87 | WNW | WSW | | Fine | Fine |
| 25 | | | 50 | 52 | 49 | 29.88 | 29.95 | 82 | 84 | W | WSW | | | |
| 26 | | | 51 | 52 | 48 | 30.00 | 29.97 | 86 | 87 | SW | SW | | | |
| 27 | | | 50 | 52 | 39 | 29.70 | 29.70 | 86 | 88 | W | W | | | Rain |
| 28 | | | 40 | 42 | 37 | 29.50 | 29.46 | 87 | 80 | SW | NW | Cloud. | Sho'ry | |
| 29 | | | 39 | 40 | 30 | 29.60 | 29.86 | 76 | 76 | NW | NW | Fair | Fair | |
| 30 | | | 32 | 37 | 35 | 29.98 | 29.97 | 75 | 80 | WNW | SW | Foggy | Fine | Fine |
| 31 | ● | | 32 | 37 | 29 | 29.90 | 29.86 | 80 | 80 | SSW | S | Fine | | |
| Feb. | | | | | | | | | | | | | | |
| 1 | | | 32 | 39 | 30 | 29.72 | 29.85 | 77 | 80 | S | S | | | |
| 2 | | | 32 | 39 | 35 | 29.94 | 30.02 | 78 | 81 | ESE | S | Foggy | Fair | Foggy |
| 3 | | | 34 | 40 | 40 | 29.87 | 29.83 | 81 | 85 | S | SSW | | | Fair |
| 4 | | | 44 | 44 | 38 | 29.60 | 29.63 | 89 | 89 | WSW | SW | Fair | Fine | |
| 5 | | | 40 | 43 | 36 | 29.71 | 29.80 | 82 | 85 | W | SSW | Fine | | |
| 6 | | | 35 | 44 | 35 | 29.92 | 30.00 | 81 | 85 | W | SW | Fair | | |
| 7 | | | 45 | 50 | 48 | 30.02 | 30.06 | 87 | 94 | SW | SW | Rain | Rain | Rain |
| 8 | ☽ | | 51 | 51 | 49 | 30.15 | 30.25 | 90 | 92 | SW | WSW | Overc. | Cloud. | |
| 9 | | | 47 | 52 | 45 | 30.25 | 30.40 | 94 | 94 | WSW | N | | Rain | Cloud. |
| 10 | | | 41 | 52 | 40 | 30.25 | 30.10 | 90 | 90 | S | W | | Fair | Fair |
| 11 | | | 41 | 46 | 36 | 30.20 | 30.15 | 67 | 72 | WNW | W | Fine | Fine | |
| 12 | | | 41 | 47 | 38 | 29.90 | 29.46 | 93 | 95 | SW | WSW | Rain | Cloud. | Sho'ry |
| 13 | | | 40 | 45 | 37 | 29.18 | 28.85 | 80 | 97 | SW | E | Fine | Rain | Rain |
| 14 | ○ | | 37 | 44 | 40 | 28.80 | 29.10 | 100 | 95 | S | N | Rain | Fair | Fine |
| 15 | | | 36 | 42 | 31 | 29.35 | 29.50 | 77 | 80 | NE | N | Fine | Fine | |
| 16 | | | 32 | 40 | 32 | 29.47 | 29.40 | 81 | 80 | NNE | SE | Fair | | |
| 17 | | | 35 | 40 | 32 | 29.30 | 29.25 | 78 | 82 | ESE | E | Foggy | Fair | Fair |
| 18 | | | 37 | 45 | 35 | 29.17 | 29.30 | 89 | 85 | E | SE | Rain | Chang. | Rain |
| 19 | | | 36 | 46 | 38 | 29.27 | 29.30 | 80 | 80 | E | E | Fair | Fine | Fair |

**We are unable to give the quantity of Rain fallen this month, on account of a leak in the Rain-guage.**

